# Diffusion tension imaging is a good tool for assessing patients with dementia and behavioral problems and discriminating them from other dementia patients

**DOI:** 10.1177/20584601211066467

**Published:** 2021-12-17

**Authors:** Mala Naik, Morteza Esmaeili, Owen Thomas, Jonn T Geitung

**Affiliations:** 1 Department of Geriatrics, 72982 Haraldsplass Deaconess Hospital, University of Bergen, Bergen, Norway; 2 Department of Research Support, Section of Statistics, 60483 Akershus University Hospital, Nordbyhagen, Norway; 3 Department of Radiology, 60483 Akershus University Hospital, Nordbyhagen, Norway; 4 Institute of Clinical Medicine, 6305 University of Oslo, Oslo, Norway

**Keywords:** Dementia, Alzheimer’s disease with and without behavioral changes, MRI and measuring fractional anisotropy

## Abstract

**Background:**

Dementia is one of the leading public health concerns as the world’s population ages. Although Alzheimer’s disease (AD) is the most common dementia diagnosis among older patients, some patients have additional behavioral symptoms. It is therefore important to provide an exact diagnosis, both to provide the best possible treatment for patients and to facilitate better understanding.

**Purpose:**

To investigate whether magnetic resonance imaging (MRI) with fractional anisotropy (FA) can accurately find patients with behavioral symptoms within a group of AD patients.

**Material and Methods:**

Forty-five patients from the geriatric outpatient clinic were recruited consecutively to form a group of patients with AD and behavioral symptoms (AD + BS) and a control group of 50 patients with established AD. All patients had a full assessment for dementia to establish the diagnosis according to ICD-10. MRI included 3D anatomical recordings for morphometric measurements, DTI for fiber tracking, and quantitative assessment of regional white matter integrity. The DTI analyses included computing of the diffusion tensor and its derived FA index.

**Results:**

We found a significant difference in FA values between the patient groups’ frontal lobes. The FA was greater in the study group in both left (0.39 vs 0.09, *p* < 0.05) and right (0.40 vs 0.16, *p* < 0.05) frontal lobes.

**Conclusion:**

MRI with FA will find damage in frontal tracts and may be used as a diagnostic tool and be considered a robust tool for the recognizing different types of dementia in the future.

## Introduction

Dementia is one of the leading public health concerns as the world’s population ages. The number of diagnosed patients varies throughout the world, possibly due to methodological differences such as the different criteria used for diagnosing dementia, different ways of data collection, sampling methods, and the different age structures of each population.^1,2^ More precise diagnostics and ongoing work are needed.^3^ It is important to achieve an exact diagnosis, to provide the best possible treatment for patients and facilitate understanding.

In addition to clinical tests, diagnostic methods such as imaging and genetics are used.^4,5^ The imaging procedures in question are magnetic resonance imaging (MRI) with volumetry of the hippocampus,^6,7^ perfusion, and MR spectroscopy,^8^ as well as diffusion tensor imaging (DTI),^4^ and nuclear medicine with PET and SPECT.^9^ MR volumetry of the hippocampus is most widely used.^6,7^ The use of MRI with DTI, with or without fiber tracking is relatively new,^4^ but it has become widely used in brain research. Several investigators have shown that damage to white matter (WM) in AD is not apparent with conventional imaging. In addition, white matter lesions in Alzheimer’s disease (AD) can be detected with DTI even before the gray matter injury becomes apparent, which assists in diagnosis and disease monitoring.^10^ The specific regions of alterations in diffusion and anisotropic diffusion vary from one study to another. Some authors report anterior differences and others posterior or temporal changes.^11,12^ In patients with AD, decreased fractional anisotropy (FA) values have been reported in the WM of the temporal^11,12^ and parietal^13^ lobes, hippocampus,^13^ superior longitudinal fascicles,^13,14^ cingulate gyrus, and corpus callosum.^12^ These conflicting results may be due to variation in the selection of patient populations.

Diffusion tensor imaging with quantitative fiber tracking might enable the assessment of age-related degeneration in the central nervous system.^15^ DTI is a useful tool in both early detection and monitoring of disease progression.^14,16,17^ DTI measures the diffusion of water through the brain tissue and can be used to visualize and measure the integrity of white matter tracts.

Patients with AD have a reduced FA in white matter, as measured with DTI.^14,16,17^ Several studies have shown a correlation between aging and changes in the white matter as well as a correlation between DTI findings and cognitive decline.^18–20^ We know that FA reduces with increasing age. However, it is not fully established whether diseases such as frontal lobe dementia show a specific reduction in FA, although two articles have presented results indicating this.^19,20^ It is of further interest to establish whether a reduction in FA frontally correlates with the degree of frontal lobe symptoms. One study has shown for the first time that the integrity of specific white matter tracts is a mediator of age-related changes in cognitive performance.^19^ In this article, we investigate whether MRI with FA can be of use in differential diagnoses for people with AD and behavioral symptoms (frontal lobe symptoms). By investigating the FA of all lobes, comparing patients both with and without behavioral symptoms, we will see how much information we can gain from MRI with FA. This investigation will evaluate the FA values of the WM tracts in patients with dementia, with and without frontal lobe symptoms, and look for a relation between frontal lobe symptoms and reduced FA in white matter/fascicles in the frontal lobes. If so, it may be a valuable tool in more precise examinations of dementia patients.

This investigation aims to explore whether MRI with fractional anisotropy can accurately diagnose patients with behavioral symptoms within a group of AD patients.

## Material and methods

Ninety-five patients were examined with the inclusion criteria of a clinically diagnosed dementia with and without frontal lobe symptoms. All patients had a full assessment for dementia to establish the diagnosis according to the International Classification of Diseases 10 (ICD-10).^21^ Forty-five patients from the geriatric outpatient clinic were recruited consecutively in the study group of patients with AD and behavioral symptoms and a control group of 50 patients with established AD, but without behavioral symptoms, giving 95 patients. The two groups were matched as well as possible: with a mean age of 76 vs 75 years. The criteria that were used to include the study patients were dementia with loss of initiative, inhibition, inattentiveness, inability to concentrate, behavior disorders, difficulty in learning new information, and inappropriate social behavior.

The following psychometric tests were performed:

1. The Mini-Mental State Examination (MMSE) or Folstein test^22^ which is a brief 20-item questionnaire test with a score range of 0–30 used to assess cognition. The scale tests six areas of cognitive function: orientation, registration, attention, calculation, recall, and language. The MMSE was used to screen all patients and control for evidence of dementia.
2. The clock-drawing test (CDT)^23^ assesses executive, visuospatial, and visuoconstructive functions.
3. The Frontal Behavioral Inventory or Frontal Lobe Inventory (FLI)^24^ is a 24-question scale, which is based on a caregiver interview assessing the patients’ behavioral problems.
4. Neuropsychiatric Inventory (NPI)^25^ is a questionnaire to assess ten behavioral disturbances occurring in dementia patients: delusions, hallucinations, dysphoria, anxiety, agitation/aggression, euphoria, disinhibition, irritability/lability, apathy, and aberrant motor activity. The NPI uses a screening strategy to assess the frequency and severity of each behavior. Information for the NPI was obtained from a caregiver familiar with the patient’s behavior.

Radiological examination: The MRI examination was performed on a GE Optima 1.5T scanner (GE Healthcare, Milwaukee, Wisc., USA) with a 24-channel head coil: 2D EPI/SE sequence, auto shim, TR= 7900 ms, TE = 97 ms, flip angle= 90°, 26 axial contiguous 4 mm slices, a field of view of 240 mm, 128 × 128 acquisition matrix, five b = 0 acquisitions and 25 diffusion sensitizing directions with b = 1000 s/mm^2^, two averages, and a total scan time of 8–10 min.

**Figure 1.**
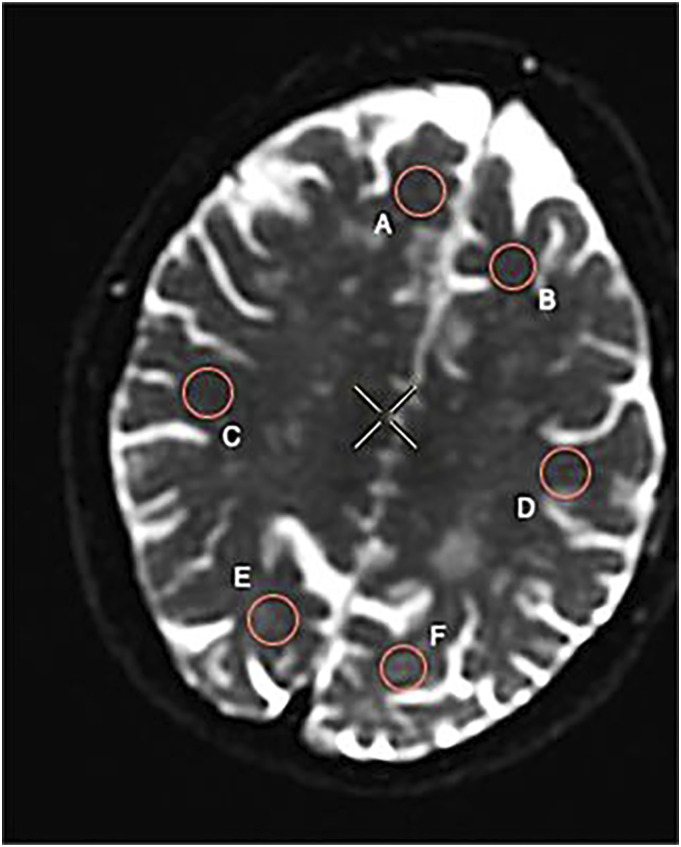
Fractional anisotropy (FA) with regions of interest (ROIs) displayed for one of our patients: (A) Right frontal lobe; (B) left frontal lobe; (C) right temporal lobe; (D) left temporal lobe; (E) right occipital lobe; and (F) left occipital lobe.

**Figure 2.**
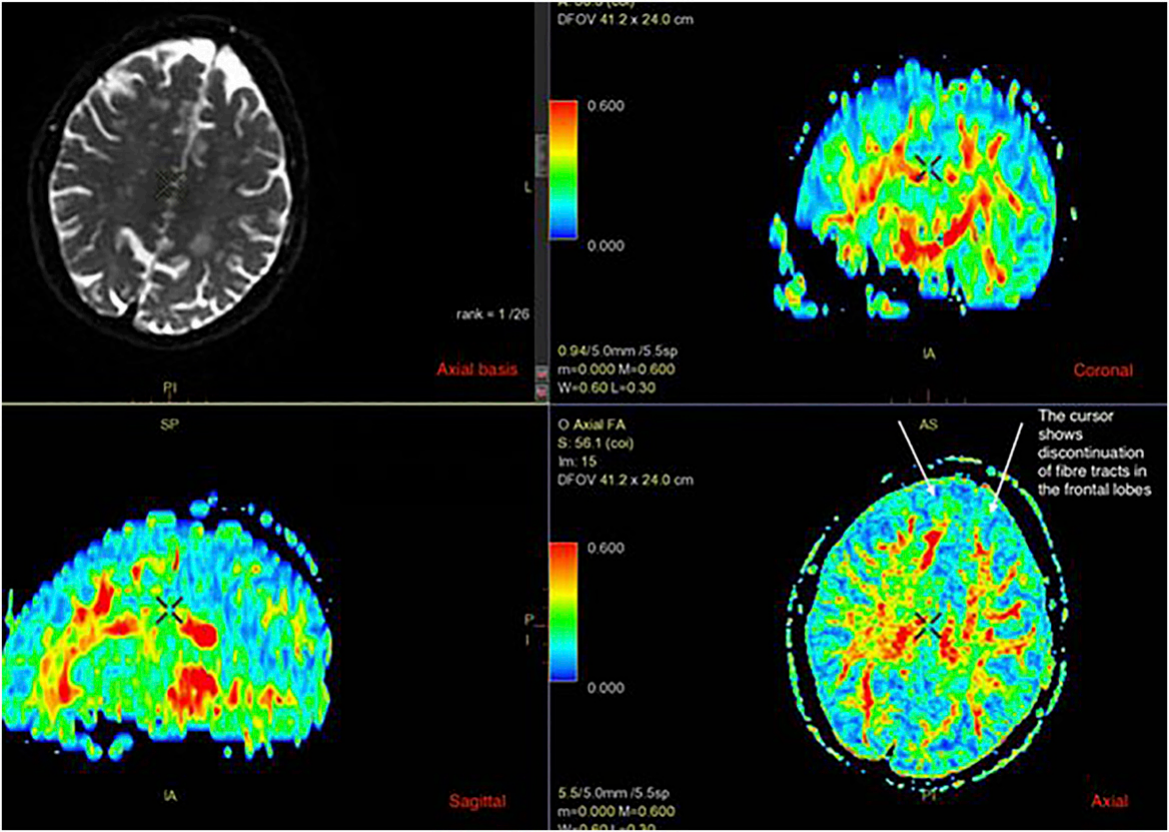
An example of diffusion tensor imaging MRI in a patient with dementia.

The pulse sequences include 3D anatomical recordings for morphometric measurements, DTI for fiber tracking, and quantitative assessment of regional white matter integrity. The DTI analyses included computing the diffusion tensor and its derived FA index. FA values were computed for each subject in the left and right frontal lobes and temporal and occipital lobes: Figures 1 and 2 show where the regions of interest (ROIs) were placed.

The readings and ROI placements were initially done by two observers independently (MN and JTG) and then as a consensus. Next, both experts computed the FA values over defined voxels within the agreed ROIs using the vendor’s post-processing tools. We performed statistical analysis on independent report of FA values. All clinical tests were correlated to the DTI results. We performed logistic regression analyses with lasso penalization, trained using 10-fold cross-validation, in order to analyze the results. The presence or absence of behavioral problems was used as a binary response variable, and the covariates were the FA values from the frontal, temporal, and occipital lobes averaged across hemispheres, together with the MMSE and CDT, to determine whether the two groups had appropriately matched levels of dementia. The lasso penalization will have the effect of shrinking small parameter values to exactly zero, providing parsimonious and interpretable maximum likelihood estimate values. The strength of the penalization was established using 10-fold cross-validation. The covariates were normalized to have zero mean and unit variance to enable direct interpretation of the scale of the β parameters of the regression model.

All subjects included in the study signed informed written consent to participate in the study. The Regional Committee approved the study for Medical Research Ethics.

## Results

The overall results of the clinical psychometric tests are presented in Table 1. The overall results of FA measurements are presented in Table 2, and the results of the lasso-penalized logistic regression are presented in Table 3. We found a significant difference in FA between the patient groups’ frontal lobes. The FA difference between left and right frontal lobes was 0.09 vs 0.39 and 0.16 vs 0.40. This was a significant difference (Table 2) between the study group and the control group (*p* < 0.05).

**Table 1.** Results of cognitive testing for the two groups. These are the average results from the tests; the two groups must be as close as possible for MMS and CDT in order to be comparable groups and differ as much as possible for NPI and FLI in order to confirm our hypothesis. Abbrevi*ations*: MMSE: Mini-Mental Status Examination; CDT: clock-drawing test; NPI: Neuropsychiatric Inventory; FLI: Frontal Lobe Inventory.

	MMSE	CDT	NPI	FLI
With frontal lobe symptoms	20.1	3.2	11.7	25.0
Without frontal lobe symptoms	21.1	3.08	4.16	1.5

**Table 2.** A presentation of fractional anisotropy (FA) values in patients with and without behavioral problems. The values presented are averages of FA in right frontal lobe (RF), left frontal lobe (LF), right temporal lobe (RT), left frontal lobe (LT), right occipital lobe (RO), and left occipital lobe (LO). A chi-square test was done to look for differences between the measurements and * denotes *p* < 0.05. We did only find differences between the measurements of the frontal lobes.

Fractional anisotropy (FA)	With behavioral problems	Without behavioral problems
FA_RF	0.16	0.4*
FA_LF	0.13	0.4*
FA_RT	0.34	0.3
FA_LT	0.33	0.3
FA_RO	0.40	0.37
FA_LO	0.40	0.31

**Table 3.** Logistic regression parameters for each covariate used for predicting behavioral problem status. The lasso-regularized maximum likelihood estimates for the parameters of the logistic linear regression were used to predict the presence or absence of behavioral problems in the patients.

Covariate	Intercept	MMSE	CDT	Frontal	Temporal	Occipital
β parameter	−0.7179	0	0	−3.4058	0.07604	0

The temporal and occipital lobes had no significant differences (Table 2) between the groups, confirming both groups to be comparable concerning AD. All lobes had low values. General FA in group 1 was lower than FA (Table 2), indicating that the patients in group 1 may have a higher degree of disease. The results from the lasso-penalized logistic regression (Table 3) indicate that the frontal lobe velocity averaged between hemispheres is a powerful and robust negative predictor of behavioral problems in patients, that is, a larger frontal lobe velocity is a strong predictor of a lower incidence of behavioral problems. A weak positive association was found between temporal lobe velocity and behavioral problems, but this is a factor of 50 weaker than the frontal lobe association and as such is likely to be a relic of the finite amount of data. The regression parameters associated with the MMSE and CDT were shrunk to exactly zero by the penalization, indicating that the dementia scores do not function as predictors of the behavioral problems, and such the dementia levels of the two groups are appropriately matched and are not confounding the results. The correlation between FA and frontal lobe inventory showed that there was a significant correlation between frontal lobe symptoms and reduced FA in the frontal lobes (Table 2). The group with frontal lobe symptoms had a generally lower FA than the patients with AD without frontal lobe symptoms.

## Discussion

This investigation demonstrated that measured frontal lobe FA may serve as a robust predictor of frontal lobe symptoms. This predictive ability was present even when including the potential confounder of dementia severity. We have a control group of patients with Alzheimer dementia without frontal lobe symptoms. Both groups were recruited as consecutive patients and could thus not be comparable. When matching age and some other aspects, group 1 presented with a more severe AD. It is a weakness that the study group was worse on the clinical testing and as expected had a generally lower FA. It is known that with lower MMSE and other cognitive testing, the general FA is lower.^17,22,23^ However, we registered a difference in FA in the frontal lobes large enough to be an indication of differences in diagnoses and not only severity of the disease. Another aspect confirming this is that the differences in the frontal lobe FA were higher than differences in other areas.

Several other articles have described the use of FA in people with dementia.^24^ However, our study is the only one that directly compares the diagnostic value of FA in patients suffering from AD with behavioral symptoms to those AD patients without behavioral problems. The other articles approaching this subject also did not include a large number of patients. Recruiting patients with dementia is a major challenge, especially with frontal lobe symptoms, and we believe this study to have one of the largest patient populations presented. An additional problem is to find subgroups of AD, in our case with behavioral symptoms. This study indicates that the methods considered may be a tool for differentiating the two conditions.

Our functional MRI method is fairly short and simple to perform. It needs some expertise to use it as a diagnostic tool, but clinics diagnosing and treating people with dementia ought to have such expertise. The sequence only requires five extra minutes in the MRI and is a short and effective way to achieve a more exact diagnosis of frontal lobe symptoms, compared to using elaborate PET or several other tools.

We used a commercially available tool for diagnosing FA. In research protocols, we usually see methods that are more refined and require more time and expertise. We believe we have demonstrated that the MRI method is robust enough for using available and practical tools for finding FA values. This study has demonstrated a way of distinguishing two groups of patients in a simple way, that is, dementia patients with or without frontal lobe symptoms.

Magnetic resonance imaging with FA will find damage in frontal tracts and may be used as a diagnostic tool and be considered a robust tool for diagnosis. DTI with FA may accordingly become a tool in the identification of different types of dementia in the future.
